# Ferroptosis in inflammatory arthritis: A promising future

**DOI:** 10.3389/fimmu.2022.955069

**Published:** 2022-07-26

**Authors:** Siyuan Chang, Mengshi Tang, Bikui Zhang, Daxiong Xiang, Fen Li

**Affiliations:** ^1^ Department of Rheumatology and Immunology, The Second Xiangya Hospital of Central South University, Changsha, China; ^2^ Department of Pharmacy, The Second Xiangya Hospital of Central South University, Changsha, China

**Keywords:** ferroptosis, inflammatory arthritis, iron accumulation, lipid peroxidation, cell death

## Abstract

Ferroptosis is a kind of regulatory cell death (RCD) caused by iron accumulation and lipid peroxidation, which is characterized by mitochondrial morphological changes and has a complex regulatory network. Ferroptosis has been gradually emphasized in the pathogenesis of inflammatory arthritis. In this review, we summarized the relevant research on ferroptosis in various inflammatory arthritis including rheumatoid arthritis (RA), osteoarthritis, gout arthritis, and ankylosing spondylitis, and focused on the relationship between RA and ferroptosis. In patients with RA and animal models of RA, there was evidence of iron overload and lipid peroxidation, as well as mitochondrial dysfunction that may be associated with ferroptosis. Ferroptosis inducers have shown good application prospects in tumor therapy, and some anti-rheumatic drugs such as methotrexate and sulfasalazine have been shown to have ferroptosis modulating effects. These phenomena suggest that the role of ferroptosis in the pathogenesis of inflammatory arthritis will be worth further study. The development of therapeutic strategies targeting ferroptosis for patients with inflammatory arthritis may be a promising future.

## 1 Introduction

Ferroptosis is a kind of regulatory cell death (RCD) that was firstly proposed by Stockwell’s team in 2012 (1) and officially defined by the Cell Death Nomenclature Committee in 2018. In simple terms, the mechanism of ferroptosis is mainly the cellular dysfunction resulting from lipid peroxidation caused by excessive intracellular iron accumulation, and abnormal mitochondrial structure is its manifestation. The occurrence and development of various diseases are affected by ferroptosis, including neurodegenerative diseases, tumors, and organ damage caused by ischemia-reperfusion such as acute renal failure, rhabdomyolysis, and heart damage (2). Ferroptosis has been studied as a potential drug target in the field of tumor therapy. Even though research about how ferroptosis is involved in disease pathogenesis and drug development based on it is in full swing, research in inflammatory arthritis and ferroptosis is still in its infancy. In recent years, some studies have shown that ferroptosis was related to the development of rheumatoid arthritis (RA) and other inflammatory arthritis. This article reviewed related studies on the relationship between ferroptosis and inflammatory arthritis, especially RA, whose purpose is to link the past and the future and cause the research interest in ferroptosis in inflammatory arthritis.

## 2 The overview of ferroptosis

Ferroptosis is a kind of RCD distinct from apoptosis, necrosis, and autophagy, each of which has unique characteristic morphological features at the cellular and subcellular levels. In the initial reports, instead of changes in the membrane, cytoplasm, and nucleus, the morphological changes of ferroptotic cells were mainly exhibited in mitochondria, including increased mitochondrial membrane density, decreased mitochondrial volume, reduced or disappeared mitochondrial cristae, and rupture of the mitochondrial outer membrane (OMM) (3). These were considered the main characteristics of distinguishing ferroptosis from other RCD. The detection of typical mitochondrial morphological changes using transmission electron microscopy (TEM) represented one of the commonly available methods for the identification of ferroptosis (4). In addition to the morphological changes that occur in mitochondria, cell membrane rupture caused by lipid peroxidation was also described during ferroptosis (5).

**Figure 1:**
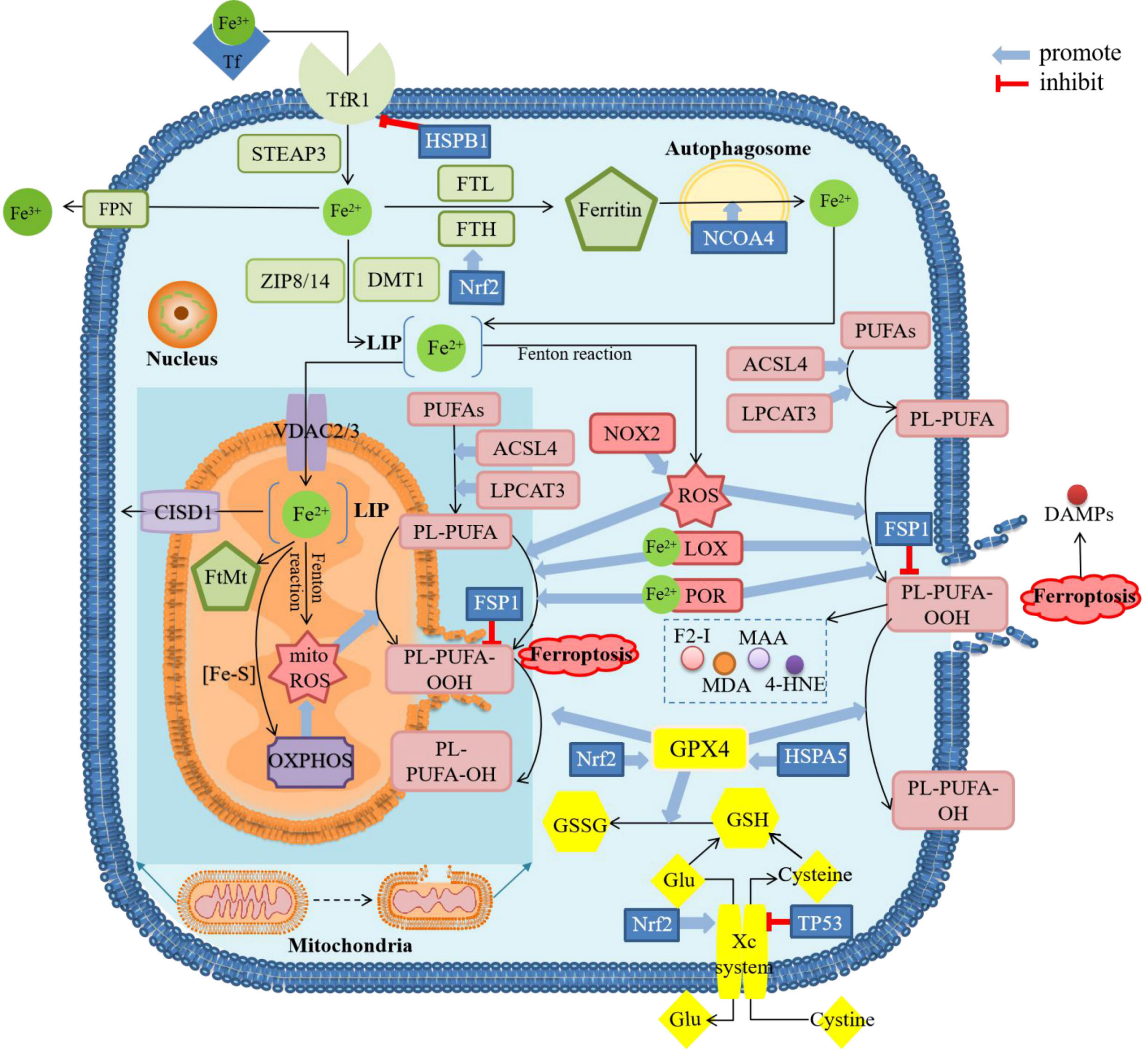
Diagram about mechanism of ferroptosis. ACSL4, acyl-CoA synthetase long-chain family member 4; CISD1, CDGSH iron sulfur domain 1; DAMPs, damage-associated molecular patterns; DMT1, divalent metal transporter 1; F2-I, isoprostane; FPN, ferroportin; FSP1, ferropsis -suppressor-protein 1; FTH, ferritin heavy chain; FTL, ferritin light chain; FtMt, mitochondrial ferritin; Glu, glutamate; GSH, glutathione; GSSG, oxidized glutathione; GPX4, glutathione peroxidase 4; 4-HNE, 4-hydroxynonenal; HSPA5, heat shock protein family A member 5; HSPB1, heat shock protein beta-1; LOX, lipoxygenase; LPCAT3, lysophosphatidylcholine acyltransferase 3; MAA, malondialdehyde-acetaldehyde; MDA, malonaldehyde; mitoROS, mitochondrial reactive oxygen species; NCOA4, nuclear receptor coactivator 4; NOX2, nicotinamide adenine dinucleotide phosphate (NADPH)-dependent oxidase2; Nrf2, nuclear factor erythroid 2-related factor 2; OXPHOS, oxidative phosphorylation; PL, phospholipids; POR, cytochrome P450 oxidoreductase; PUFAs, polyunsaturated fatty acids; ROS, reactive oxygen species; STEAP3, six-transmembrane epithelial antigen of the prostate 3; TfR1, transferrin receptor 1; VDAC, voltage-dependent anion channels; ZIP8/14, Zinc-Iron regulatory protein family 8/14. There are four main sections in the figure showing the mechanism of ferroptosis. The green squares mainly show the metabolic process of iron after entering the cell, including reduction, storage and release, and then active iron becomes an important factor in promoting lipid peroxidation. The pink squares show the process of lipid peroxidation, including the binding of PUFAs and PL, followed by the peroxidation of this complex by ROS and the release of downstream products. The yellow squares show the role of the cellular antioxidant system. GPX4 is the only enzyme that can reduce lipid peroxides, whose inactivation is thought to be the central part of ferroptosis. The content in the dark blue background shows the major changes in mitochondria during ferroptosis, including morphological changes and biochemical reactions. These processes can be modulated by several regulatory factors. The result of ferroptosis is cell rupture and the release of DAMPs.

The physiological activities in the process of ferroptosis can be mainly divided into two parts, namely iron accumulation and lipid peroxidation which is regarded as the direct cause of cell death (**Figure 1**). As the key enzyme in cellular resistance to lipid peroxidation, it is widely believed that the inactivation of glutathione peroxidase 4 (GPX4) is the prerequisite for ferroptosis. In addition to the classical ferroptosis pathway with GPX4 as the core described by Stockwell, some studies also proposed GPX4-independent pathways, such as ferroptosis regulated by p53 (6). As an increasing number of studies have confirmed that ferroptosis is closely related to the occurrence of various diseases, more research about the detailed mechanism of ferroptosis is urgently needed.

### 2.1 Important process of ferroptosis

#### 2.1.1 Iron accumulation

Ferric ions (Fe^3+^) bind to transferrin (TF) in the blood circulation and the complex can then be transferred into the endosomes inside the cells *via* transferrin receptor 1 (TfR1) on the cell membrane. After being dissociated from the complex and converted from Fe^3+^ by six-transmembrane epithelial antigen of the prostate 3 (STEAP3), divalent ferrous ions (Fe^2+^) in the endosomes are transferred into the cytoplasm by divalent metal transporter 1 (DMT1) or Zinc-Iron regulatory protein family 8/14(ZIP8/14). In the cytoplasm, a small portion of Fe^2+^ remains in the labile iron pool (LIP), and the majority of Fe^2+^ is combined with ferritin light chain (FTL) and ferritin heavy chain 1 (FTH1) to form ferritin for storage. Excess Fe^2+^ can be transported to the outside of the cell and converted to Fe^3+^ by ferroportin (FPN, also known as SLC40A1) to maintain the balance of iron in the cytoplasm.

In addition to dysregulation of proteins associated with LIP maintenance in the cytoplasm which will induce iron accumulation, another source of active Fe^2+^ is autophagy of ferritin with the help of nuclear receptor coactivator 4 (NCOA4) (3). Derived from the above two major pathways, accumulation of active Fe^2+^ will produce reactive oxygen species (ROS) through the Fenton reaction or directly catalyze lipid peroxidation as a cofactor of lipoxygenase (LOX) (7), which can both induce the occurrence of ferroptosis.

#### 2.1.2 Lipid peroxidation

Two important metabolites of lipid peroxidation are ROS and lipids. As one of them, ROS will be mostly produced through the Fenton reaction and two other ways, including oxidative metabolism, occurred in the mitochondria and enzymatic reactions performed by nicotinamide adenine dinucleotide phosphate (NADPH)-dependent oxidase (NOX) family (8).

As for another important metabolite, polyunsaturated fatty acids (PUFAs) represented by arachidonic acid (AA) are the main substrates for lipid peroxidation during ferroptosis. Before being peroxidized, PUFAs are thioesterified by acyl-CoA synthetase long-chain family member 4 (ACSL4) and then combined with phospholipids to integrate into the plasma membrane by lysophosphatidylcholine acyltransferase 3 (LPCAT3), finally polyunsaturated fatty acyl moieties present on phospholipids (PL-PUFAs) are formed. PL-PUFAs are involved in the downstream ferroptosis process (3, 9, 10) as the direct substrate for lipid peroxidation, which can occur either non-enzymatically or enzymatically. In the non-enzymatic pathway, the hydroxyl radicals (HO·) generated through the Fenton reaction involving Fe^2+^ can deprive lipids of H· to form lipid radicals, which then form lipid peroxide, resulting in non-enzymatic lipid peroxidation (11). In the enzymatic pathway, at least two pathways have been identified, one of which is that iron-dependent LOXs can catalyze the formation of PUFAs into peroxides and the formation of its derivatives, such as malonaldehyde (MDA) and 4-hydroxynonenal (4-HNE), etc. (8). As for another enzymatic pathway, cytochrome P450 oxidoreductase (POR) has been confirmed to promote lipid peroxidation leading to ferroptosis (12), which requires iron-catalysis (13). Lipid peroxidation can lead to the dysfunction of enzymes on the membrane and eventually change the fluidity and permeability of the plasma membrane (14).

While lipid peroxidation occurs, cells employ antioxidant systems to combat this process and maintain balance which is mainly composed of cystine/glutamate antiporter (System Xc-) and GPX4. The System Xc- which consists of light chain solute carrier family 7 member 11 (SLC7A11, xCT) and heavy chain solute carrier family 3 member 2 (SLC3A2) can transport glutamate (Glu) out of the cell and cystine into the cell. Cystine will be then transformed into cysteine and become the substrate for glutathione (GSH) synthesis. GPX4 is the only enzyme with this function of converting GSH to oxidized glutathione (GSSG) and reducing lipid peroxides to the corresponding alcohol at the same time (7), hence GPX4/SLC7A11 is regarded as the key regulating factor for ferroptosis.

#### 2.1.3 The role of mitochondria in ferroptosis

The researches on the role of mitochondria in ferroptosis were extensive but not systematic. The involvement of mitochondria in ferroptosis as an important source of ROS is still the current mainstream view, since mitochondrial ROS (mitoROS) is mainly produced by oxidative phosphorylation (OXPHOS) complexes in mitochondria (15). MitoROS can lead to lipid peroxidation of the mitochondrial membrane (16), which is supported by the fact that the subcellular localization of lipid-ROS occurred first in the mitochondrial distribution in cystine-starvation-induced and Erastin-induced ferroptosis (17). In addition to promoting ferroptosis by producing ROS, mitochondria appear to inhibit ferroptosis in other ways, for example, mitochondria may be involved in ferroptosis by consuming Glu through the tricarboxylic acid (TCA) cycle to resist the inhibitory effect of high extracellular Glu on the Xc- system (11).

Although mitochondria are closely related to ROS production and iron metabolism, the causal relationship between morphological changes of mitochondria and ferroptosis remains unclear. In cystine-starvation-induced and xCT-inhibition-induced ferroptosis, mitochondrial rupture, mitoROS production, and mitochondrial membrane potential (MMP) loss were observed, whereas these phenomena were not observed in GPX4 inhibitor-induced ferroptosis (17, 18). More research may be needed to explore the profound mechanism of mitochondria in ferroptosis.

### 2.2 Consequence of ferroptosis

Some studies suggested that ferroptosis was a form of immunogenic cell death (ICD) (19), as many products of ferroptosis were proved to be mediators of inflammation. Firstly, lipid peroxides are unstable whose derivatives can not only act as inflammatory mediators but also react with proteins and make them antigenic (20). For example, 4-HNE, as one of the derivatives of lipid peroxide, could activate Src (a non-receptor tyrosine kinase) which in turn activates inflammatory signaling pathways such as the nuclear factor κB (NF-κB) pathway in the aged kidney (21). In addition to lipid peroxides, damage-associated molecular patterns (DAMPs) released by ferroptotic cells such as cell-free DNA (cfDNA) (22) and high mobility group 1(HMGB1) are also important mediators of inflammation (23). HMGB1 could act as an adjuvant to activate both the natural and acquired immune systems, which can upregulate the secretion of tumor necrosis factor α (TNF-α) from macrophages and take part in the autophagy-related gene (ATG) 5-and ATG7-dependent programmed cell death (PCD) (23).

### 2.3 Regulators of ferroptosis

Ferroptosis regulators perform in various manners, including iron uptake, iron export, and antioxidation. Among the regulators of iron metabolism, iron metabolic regulating proteins (IRPs), iron responsive element (IRE), heat shock protein beta-1 (HSPB1), and hepcidin were regarded as potential regulators of ferroptosis. The interaction of IRPs and IRE could promote the expression of TfR1 and inhibit the expression of FTH and FPN1 (24), and hepcidin could cause degradation of FPN (25), both of which could lead to the accumulation of intracellular iron. HSPB1 could inhibit the expression of TfR1 so it was considered as an inhibitor of ferroptosis (3, 4, 7). Among the regulators of lipid peroxidation, another member of the heat shock protein family, heat shock protein family A member 5 (HSPA5) that can stabilize GPX, was also an inhibitor of ferroptosis (3, 4, 7). Apoptosis-inducing factor-mitochondria-associated 2 (AIF-M2) which is also called ferroptosis-suppressor-protein 1 (FSP1) could indirectly inhibit lipid peroxidation by catalyzing the regeneration of coenzyme Q10 (CoQ10), an antioxidant (26). The nuclear factor erythroid 2-related factor 2 (Nrf2) regulated by Kelch-like ECH-associated protein-1 (Keap1) could activate antioxidant response elements (ARE) including FTH1, heme oxygenase 1 (HO-1), and NAD(P)H: quinone oxidoreductase (NQO1), to inhibit ferroptosis, which was called Keap1-Nrf2-ARE pathway (27).

The tumor suppressor protein p53 (TP53), a well-known transcription factor in the tumor, has been proved to play a dual role in regulating ferroptosis. About the pathway by which p53 promotes ferroptosis, it could not only regulate the expression of the downstream proteins glutaminase 2 (GLS2) (28) and arachidonate 15-lipoxygenase (ALOX15) (29) but also promote ferroptosis through GPX4-dependent pathway (SLC7A11/Glu/GSH) (30) or GPX4-independent pathway (p53-SLC7A11-ALOX12 [arachidonate 12-lipoxygenase]) (31). About the pathway by which p53 resists ferroptosis, the following three proteins were involved, including dipeptidyl peptidase 4 (DPP4) (32), cyclin-dependent kinase inhibitor 1A (also known as p21^WAF1/Cip1^, CDKN1A/p21) (33) and calcium-independent phospholipase (iPLA2β) (34). Although most of these results are from tumor cells, they may provide a reference for ferroptosis in other cells.

## 3 The role of ferroptosis in inflammatory arthritis

Inflammatory arthritis refers to inflammatory diseases occurring in human joints and surrounding tissues caused by inflammation, infection, trauma, or other factors. They have many common characteristics, including swelling, pain, dysfunction, and joint deformity, which can lead to disability and affect the quality of patients’ life in severe cases. In addition to RA, other common inflammatory arthritis includes osteoarthritis (OA), gout arthritis (GA), ankylosing spondylitis (AS), etc. Currently, many researchers have noticed the potential relationship between inflammatory arthritis and ferroptosis (**Table 1**), which may shed light on understanding the pathogenesis of inflammatory arthropathy and uncovering future therapeutic targets.

**Table 1:** Evidence of ferroptosis in inflammatory arthritis.

Disease	Model	Evidence of Ferroptosis	
		Evidence of iron accumulation	Evidence of lipid peroxidation/oxidative stress
RA	Patient	1. Iron deposited in synovium (35) 2. FTH, FLH, and DMT1 were positive in FLSs and macrophages; TfR were positive in FLSs (36) 3. sTfR increased, iron decreased in serum (37) 4. Iron increased, TfR1 and NCOA4 increased, Nrf2 decreased in LPS-treated synoviocytes (38) 5. Iron increased in the synovial fluid of patients with high disease activity compared with patients with moderate disease activity (39).	1. MitoROS increased in blood and monocytes (20) 2. GSH and GPx decreased in blood (40) 3. MDA increased in blood, RBC and synovial fluid; TBARS increased in blood; F2-I increased in plasma; MAA adducts increased in synovium (20, 39); 4. MDA increased, GPX4, SLC7A11 and SLC3A2L decreased in LPS-treated synoviocytes (38) 5. 4-HNE increased in the hyperplastic synovium of RA patients compared with OA patients (39).
	Animal	1. FTH1 increased in the synovium and FLSs of CIA model mice (41).	1. 4-HNE increased in the synovium of CIA model mice compared with normal mice (39). 2. ACSL4 decreased, GPX4 and SLC7A11 increased in the synovium and FLSs of CIA model mice (41).
OA	Patient	1. Iron increased in synovial fluid (42) 2. Ferritin increased in serum (43)	1. 4-HNE/protein adduct increased in synovial fluid and osteoblasts (44–47)
	Animal	1. TfR1 and DMT1 increased, FPN decreased in OA model mice and in IL-1β-treated or TNF-α-treated chondrocytes (42) 2. MMP-3 and MMP-13 increased, chondrogenic related proteins↓ in FAC-treated chondrocytes (42) 3. Cartilage destruction decreased in DMT1-inhibited OA model mice (42)	1. GPX4 and SLC7A11 decreased, ROS and ACSL4 increased in IL-1β-treated and FAC-treated chondrocytes, and FER-1 can inhibit these (48) 2. Cartilage degradation decreased, type II collagen increased in the FER-1-treated OA model mice (48)
AS	Patient	1. Iron overloaded in PMNs and platelets (49) 2. TfR decreased in serum (50)	1. TOS and OSI increased, TAS decreased in plasma (51) 2. SOD, CAT, and NO increased in the blood of AS patients with MetS (52) 3. AOPPs and ROS increased in serum (53)
	Animal	Not known yet.	1. GPx decreased in serum (54) 2. ROS and MDA increased, GPx activity↓in the connective tissue around the vertebral body (55)
GA	Patient	1. Ferritin increased in serum (56)	Not known yet.
	Animal	Not known yet.	1. MDA increased, GPx decreased in plasma, liver, and spleen (57)

ACSL4, acyl-CoA synthetase long-chain family member 4; AOPPs, oxidative protein products; AS, ankylosing spondylitis; CAT, catalase; CIA, collagen-induced arthritis; DMT1, divalent metal transporter 1; FAC, ferric ammonium citrate; FER-1, ferrostatin-1; F2-I, isoprostane; FLSs, fibroblast-like synoviocytes; FPN, ferroportin; FTH, ferritin heavy chain; FTL, ferritin light chain; FSP1, ferropsis -suppressor-protein 1; GA, gouty arthritis; GPx, glutathione peroxidase; GSH, glutathione; GPX4, glutathione peroxidase 4; 4-HNE, 4-hydroxynonenal; IL-1β, interleukin-1β; LPS, lipopolysaccharide; MAA, malondialdehyde-acetaldehyde; MDA, malonaldehyde; MetS, metabolic syndrome; mitoROS, mitochondrial reactive oxygen species; MMPs, matrix metalloproteinase; NCOA4, nuclear receptor coactivator 4; NO, nitric oxide; Nrf2, nuclear factor erythroid 2-related factor 2; OA, osteoarthritis; OSI, oxidative stress index; PMNs, polymorphonuclear cells; RA, rheumatoid arthritis; ROS, reactive oxygen species; SLC3A2, solute carrier family 3 member 2; SLC7A11, solute carrier family 7 member 11; SOD, markers superoxide dismutase; sTfR, soluble transferrin receptor; TAS, total antioxidant status; TBARS, thiobarbituric acid reactive substances; TfR1, transferrin receptor 1; TNF, tumor necrosis factor; TOS, total oxidative status.

### 3.1 Ferroptosis and RA

**Figure 2:**
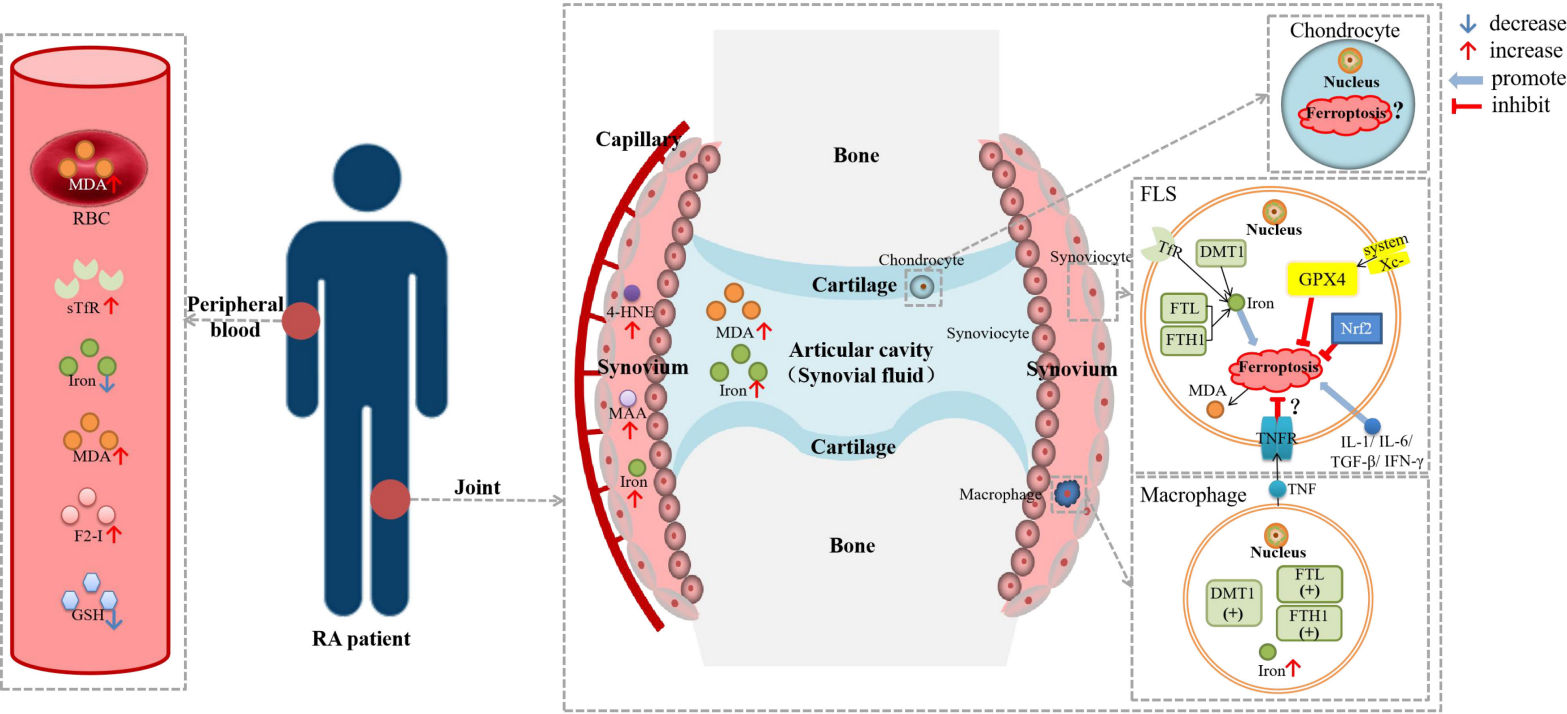
Diagram about the role of ferroptosis in RA. DMT1, divalent metal transporter 1; F2-I, isoprostane; FLS, fibroblast-like synoviocyte; FTH1, ferritin heavy chain 1; FTL, ferritin light chain; GSH, glutathione; GPX4, glutathione peroxidase 4; HSPA5, heat shock protein family A member 5; 4-HNE, 4-hydroxynonenal; IFN-γ, interferon-γ; IL-1β, interleukin-1β; IL-6, interleukin-6; MDA; malonaldehyde; NCOA4, nuclear receptor coactivator 4; Nrf2, nuclear factor erythroid 2-related factor 2; RBC, red blood cell; sTfR, soluble transferrin receptor; TGF-β, transforming growth factor-β; TNF, tumor necrosis factor; TNFR, tumor necrosis factor receptor. Lipid peroxidation were observed in the blood, synovial tissue and synovial fluid of RA patients, as well as iron deposition in the synovium. There was evidence of ferroptosis in RA-FLSs and macrophages, but it has not been determined whether ferroptosis is hyperactive or inhibited. TNF could reduce the ferroptosis sensitivity of FLSs through TNFR, while other cytokines, including IL-1, IL-6, TGF-β and IFN-γ, could promote ferroptosis in FLSs.

The main pathogenesis of RA is immune disorder and inflammation, and the main pathological changes are synovitis and progressive cartilage and subchondral bone destruction (26). Many cells are involved in the pathogenesis of RA. The upstream cells mainly include monocytes, macrophages, T cells, B cells, and neutrophils, while the downstream cells mainly contain fibroblast-like synoviocytes (FLSs), synovial macrophages, chondrocytes, etc. (58, 59). The ferroptosis of FLSs and macrophages was related to RA, and there was also some evidence that the destruction of chondrocytes may be related to ferroptosis (26) (**Figure 2**).

#### 3.1.1 Iron accumulation in RA

Researchers have found synovial iron accumulation in RA patients which may be related to the disease. As early as 1968, some researchers found iron deposition in the synovium of RA patients (35). Blake DR et al. and Roberts D et al. both observed that the input of iron-dextran could aggravate RA synovitis, of which mechanism is increasing lipid peroxidation, oxidized ascorbic acid, and decreasing GSH in red blood cells (RBCs) (60, 61). Van Vulpen et al. observed that iron overload led to synovial inflammation and hyperplasia, death of chondrocytes, and impairment of osteoblast function in hemophilia and hemochromatosis, resulting in joint destruction in all aspects (62). A study in 2018 analyzed iron homeostasis parameters (including serum iron, total iron-binding capacity [TIBC], ferritin and soluble transferrin receptor [sTfR]), inflammatory activity parameters, and autoimmune disease parameters between RA patients and healthy controls (37). The results showed that serum sTfR levels were significantly higher while serum iron levels were significantly lower in RA patients than in the control group (37). Serum iron was negatively correlated with sTfR, and sTfR was significantly positively correlated with inflammatory activity parameters and autoimmune disease parameters (37). Wu et al. observed much higher iron levels in the synovial fluid of patients with high disease activity than in that of patients with moderate disease activity (39). Iron deficiency in blood and iron accumulation in the synovium and synovial fluid of RA patients may suggest iron redistribution. Iron-deficiency anemia is not rare in RA patients, but the current recommendation is that intravenous iron should be given with great caution, as it may lead to worsening of arthralgia and synovitis (63).

Not only in tissue, but there were also abnormalities in iron metabolism in cells associated with RA, especially in downstream cells. In 2002, Telfer and Brock et al. detected FTL, FTH, and non-specific resistance-associated macrophage proteins (Nramp2, or DMT1) in FLSs and macrophages isolated from the synovium of RA patients, and TfR expression was also detected in FLSs (36). In their experiments in 2004, they also found that pro-inflammatory cytokines including interleukin-1 (IL-1), interleukin-6 (IL-6), TNF-α, and interferon-γ (IFN-γ) could increase the uptake of iron by monocytes and FLSs in RA patients *in vitro (* 64). Therefore, they speculated that inflammatory cytokines could promote the progression of RA by increasing the accumulation of iron in FLSs and macrophages in the synovium.

#### 3.1.2 Lipid peroxidation in RA

The role of ROS in RA has always attracted much attention, whose production is also one of the important processes of ferroptosis. The decrease of vitamin C and vitamin E in the serum of RA patients indicated the dysregulation of the antioxidant system (58). ROS can activate matrix metalloproteinase (MMPs), inhibit cartilage proteoglycan synthesis, and promote FLSs proliferation and chondrocyte apoptosis in RA (26, 65). Moreover, oxidative stress disorder could stimulate T cells and aggravate immune disorders (20). Researchers speculated that FSP1 could inhibit ferroptosis by inhibiting the TNF-α/ROS feedback loop to protect synoviocytes from inflammation and resist the occurrence and development of RA (26).

Researchers have been paying attention to lipid peroxidation in RA for a long time. A study that included 30 women with RA suggested that the level of MDA was increased in serum while the levels of GSH and glutathione peroxidase (GPx) were decreased in the blood in RA, which was associated with disease activity (40). According to a systematic review, several studies have analyzed the levels of lipid peroxidation markers in RA patients, showing that the levels of MDA, thiobarbituric acid reactive substances (TBARS), isoprostane (F2-I), and malondialdehyde-acetaldehyde (MAA) were elevated in different specimens (20). Lipidomic studies showed that lipid metabolism in plasma was significantly changed in RA patients, and the content of lipid peroxidation products was significantly increased (58). Luo et al. found significantly increased MDA levels in synoviocytes in the lipopolysaccharide(LPS)-induced synovitis cell model (38). As for the question of whether the activity of GPx was increased or decreased, different researchers have drawn different conclusions (20). Whether this different phenomenon is related to the characteristics of RA patients included in the studies needs more research to prove.

#### 3.1.3 Mitochondrial dysfunction in RA

Although mitochondrial dysfunction in RA has been demonstrated, it is unclear whether this phenomenon is associated with ferroptosis. Researchers found that the mitoROS levels in the blood and monocytes of RA patients were 5 times higher than that of normal people (20). It has been reported in recent years that mitochondrial dysfunction could lead to the progression of inflammation in RA. Mitochondrial dysfunction of synoviocytes could not only induce the expression of cyclooxygenase-2 (COX-2), prostaglandin E2 (PGE2), and interleukin-8 (IL-8), but also generate ROS, which triggers subsequent inflammatory responses (66). MitoROS could activate the NF-κB pathway, p38/MAPK (mitogen-activated protein kinase) pathway, and JNK1/2 (c-Jun N-terminal kinases 1/2) pathway directly or indirectly and form positive feedback with TNF-α to exacerbate the progression of RA (26). MitoROS could also activate the NOD-like receptor P3 (NLRP3) inflammasome and promote the activation of interleukin-1β (IL-1β) and interleukin-18 (IL-18) (66). DAMPs such as N-formyl polypeptides released by damaged mitochondria could bind to N-formyl peptide receptors (FPRs) to recruit neutrophils (66). Whether the mitochondrial dysfunction in RA can be associated with ferroptosis and what kind of ferroptotic cells should be linked to the mitochondrial dysfunction in RA still need further research and exploration.

#### 3.1.4 The role of ferroptosis in RA

Recently, several studies directly confirmed the involvement of ferroptosis in the pathogenesis of RA. A paper in 2021 showed that the iron content and the expression of TfR1 and NCOA4 in synovitis cells increased, while the expression of GPX4, SLC7A11, SLC3A2L, and NRF2 decreased in LPS-induced synovitis cell model, resulting in increased synovitis cells death and decreased cell viability (38). In 2022, Wu et al. found higher levels of 8-OHdG and 4-HNE in the synovium of RA patients and CIA mice than in OA patients and normal mice, as well as the higher levels of MDA and iron in synovial fluid of RA patients which were associated with disease activity (39). They also found that FLSs were more sensitive to ferroptosis than macrophages, endothelial cells, CD3+T cells or CD19+ B cells. However, some researchers have found the opposite results (39). Ling et al. found that ferroptosis decreased in the RA synovium and FLSs, with the decreased level of ACSL4 and the increased levels of FTH1, GPX4, and SLC7A11, and glycine enhanced ferroptosis *via* S-adenosylmethionine (SAM)-mediated GPX4 promoter methylation and FTH1 decrease (41). In addition, no significant ferroptosis was observed in the neutrophils of RA patients (67).These evidences suggest that more attention should be paid to the ferroptosis of cells downstream of RA.

Some indirect evidence may also suggest the involvement of ferroptosis in RA. Jing et al. found that in osteoporosis, iron overload could elevate ROS production and mitochondrial dysfunction to induce osteoblast apoptosis and promote osteoclast differentiation, which led to bone destruction (68). Although these phenomena were not linked to ferroptosis, iron overload, increased oxidative stress, and mitochondrial dysfunction all have significance for further study of the relationship between ferroptosis in osteoblast and bone destruction. As another important regulator of ferroptosis, the overexpression of p53 in FLSs was thought to be associated with increased invasiveness and bone destruction (69), but no studies linked p53 expression to ferroptosis in RA yet.

To sum up, although there were still some controversial points about whether ferroptosis levels increase or decrease in RA, ferroptosis, as a form of ICD, appeared to accelerate the disease onset and progression of RA through its pro-inflammatory effect.

However, inhibition of ferroptosis also appeared to be associated with the progression of RA. Wu et al. found that imidazole ketone erastin (IKE, a ferroptosis inducer) could decrease FLSs populations to reduce joint inflammation and destruction in CIA mice (39). And contrary to previous opinions (64), they also found that long-term exposure to TNF, which was considered to be an important cytokine in the pathogenesis and development of RA, could protect RA-FLSs from ferroptosis by promoting cystine uptake and GSH synthesis, while IL-6 and transforming growth factor-β (TGF-β) could increase the sensitivity of FLSs to ferroptosis (39). TNF could promote the proliferation of synoviocytes and lead to the progression of synovitis, and the role in reducing ferroptosis sensitivity to lessen the death of FLSs may also be a manifestation of this mechanism. From this point of view, whether ferroptosis is harmful or protective in RA is a controversial issue. Based on the current results, despite the deleterious pro-inflammatory effects of ferroptosis itself, inhibition of ferroptosis would aggravate synoviocytes proliferation and appear to cause more serious results. Therefore, how to choose drugs targeting ferroptosis in RA, such as whether to use ferroptosis inducers or inhibitors, and when to use them, still needs to be further explored.

### 3.2 Ferroptosis and OA

The pathological mechanism of OA is periarticular cartilage and subchondral bone damage and synovitis, in which chondrocytes play an important role. The iron concentration in the synovial fluid has been reported to be increased in OA patients, although it was not as significant as in RA patients (42). Higher serum ferritin levels were the indicative of increased articular radiographic severity in patients with OA than in healthy control (43). Patients with hereditary hemochromatosis, characterized by the overload of iron throughout the body, frequently suffer from OA (62). Jing et al. established an iron overload (IO) mice model by parenteral administration of iron dextran to assess the role of iron in the pathogenesis of OA (42). Compared with the control group, the IO model showed more pronounced cartilage degeneration and subchondral bone destruction and sclerosis, leading to the higher Osteoarthritis Research Society International (OARSI) scores. They observed increased ADAMTS5 (a kind of matrix-degrading enzyme) and matrix metalloproteinase-13 (MMP13) expression, as well as an increased number of osteoclasts. To explore the possible molecular mechanisms linking inflammation and iron overload in OA, they used IL-1β or TNF-α to treat chondrocytes and observed increased TfR1 and DMT1 but decreased FPN, which could lead to intracellular iron accumulation (42). Even though the results that ammonium ferric citrate (FAC)-treated chondrocytes showed increased apoptosis and MMP3 and MMP13 level links iron overload to chondrocyte apoptosis (42), it may shed light on the role of chondrocyte ferroptosis in OA. In patients with OA, 4-HNE was mainly produced in synoviocytes, and the level of 4-HNE/protein adduct in synovial fluid and osteoblasts is increased (44–46). The high level of 4-HNE was proved to be cytotoxic to human chondrocytes and osteoblasts (45, 47). Reseachers observed that mtDNA damage in OA led to chondrocyte injury (70).

A study showed that ferroptosis of chondrocytes do promote the occurrence and development of OA. Yao. et al. used IL-1β to simulate inflammation and FAC to simulate the iron overload state in chondrocytes (48). In both kinds of chondrocytes, ROS and lipid ROS accumulation, as well as the change of ferroptotic proteins including GPX4, SLC7A11, ACSL4, P53, and NRF2-ARE, could promote the occurrence of ferroptosis, while ferrostatin-1 (FER-1, a ferroptosis inhibitor) could inhibit this effect. Erastin-treated chondrocytes showed the increasing MMP13 expression but decreasing type II collagen (collagen II) expression which were considered to be markers of OA. The above conclusions were also confirmed in FER-1-treated OA model mice (48). As a kind of disease that was often analogous to RA, evidence of ferroptosis in OA, especially chondrocyte ferroptosis which might play a harmful role in OA, will not only suggest the relationship between the inflammatory microenvironment of joints and cartilage destruction but also inspire the study of chondrocyte ferroptosis in the pathogenesis of RA.

### 3.3 Ferroptosis and AS

AS is a chronic disease characterized by inflammation of spinal attachment and sacroiliac joint, leading to spinal rigidity and fibrosis and may involve the eyes, lungs, muscle, and bone. Up to now, few studies have been reported on ferroptosis in AS. In 1986, Feltelius et al. reported iron overload in polymorphonuclear cells (PMNs) and platelets in AS patients, and iron content in PMNs and platelets were negatively correlated with serum TfR levels, while iron content in PMNs was positively correlated with inflammation level (49). In 2012, a serum proteomic and metabolomic study showed that serum TfR levels were significantly down-regulated in AS patients (50).

The enhancement of oxidative stress in the pathogenesis of AS has been reported yet, although direct evidence of lipid peroxidation is still lacking in patients with AS. In 2007, some authors analyzed the total antioxidant status (TAS), total oxidative status (TOS), and oxidative stress index (OSI) in plasma of AS patients, and the results indicated dysregulation of oxidative and antioxidant systems in AS patients (51). Pishgahi et al. found that oxidative stress markers including superoxide dismutase (SOD), catalase (CAT), and nitric oxide (NO) were increased in the blood of AS patients with metabolic syndrome (MetS), but GPx and TOS levels did not change significantly (52). In 2008, Dong observed reduced serum GPx levels in a mouse AS model induced by human proteoglycan extract (54). Also in AS model mice, Feng et al. observed that the levels of ROS and MDA in the connective tissue around the vertebral body were significantly higher than those in the normal control group, and the GPx activity was significantly reduced (55). Ye et al. found that the oxidative protein products (AOPPs) and ROS content in the serum of AS patients increased, and the serum of AS patients could induce mitochondrial dysfunction in mesenchymal stem cells (MSCs) *in vitro (* 53). Although Ye et al. associated their results with cellular aging, the presence of oxidative stress and mitochondrial dysfunction will still inform the existence of ferroptosis in AS.

### 3.4 Ferroptosis and GA

GA is a chronic inflammation caused by the deposition of sodium urate crystals in joints, which leads to inflammation of joints and surrounding tissues and then results in joint swelling and pain. No direct evidence of ferroptosis in GA was shown currently, so the abnormal iron metabolism and dysregulation of the antioxidant system in GA were enumerated below. Uric acid is an antioxidant, although hyperuricemia is a prerequisite for the formation of urate crystals. Based on a nationwide population study in China, serum ferritin and TfR levels were positively correlated with serum uric acid levels and the risk of hyperuricemia (71). Serum iron/ferritin was positively correlated with the number of gout attacks, and serum ferritin was higher in gout patients than in controls (56). The mechanism by which iron is involved in gout might be related to xanthine oxidase (XO), which is responsible for the production of uric acid. Studies have shown that iron could promote the expression and activation of XO, while deferoxamine (DFO) could inhibit the activity of XO (72). Meanwhile, cytokines including TNF-α, IL-1, IFN-γ, and IL-6 could promote the activation of XO, resulting in the production of ROS (73). In addition, the researchers have proposed that iron-involved oxidative stress might also be involved in XO activation (71), which may be related to ferroptosis. In the mouse model of GA induced by sodium urate crystal, researchers observed increased levels of MDA in plasma, liver, and spleen, and decreased levels of GPx compared with the control group (57). No studies have yet linked high iron levels to lipid peroxidation during gout, and the relationship between ferroptosis and gout arthritis needs further study.

## 4 Anti-rheumatic drugs and ferroptosis

Using ferroptosis inducers to inhibit tumor progression is currently a popular direction in the field of tumor therapy. The mechanism of action of many anti-rheumatic drugs has also been confirmed to be directly or indirectly related to ferroptosis, although some conclusions were drawn from tumors (**Table 2**).

**Table 2:** Anti-rheumatic drugs with possible ferroptosis-regulating effects found in different models.

Drug		Cell line or animal model	Mechanisms regulating ferroptosis		Reference
Type	Name		Iron accumulation	Lipid peroxidation	
Glucocorticoids	DXMS	HT1080 cells	Not known yet.	Upregulation of DPEP1 to reduce GSH to increase the sensitivity to ferroptosis	(74)
		MC3T3-E1 cells; MLOY4 cells	Not known yet.	Upregulation of P53 to inhibit the expression of SLC7A11/GPX4	(75)
NSAIDs	IND	GA mouse model	Not known yet.	Upregulation of GPx activity to reduce MDA levels	(57)
	IBU	Glioblastoma cells	Not known yet.	Upregulation of Nrf2, GPX4 and SLC7A11	(76)
DMARDs	MTX	T cells	Not known yet.	Promotion of ROS production	(77)
		HT22 cells	Promotion of iron accumulation; Upregulation of FTH1 and downregulation of NCOA4	ROS accumulation	(78)
	SASP	Lymphoma cells	Not known yet.	Inhibition of System Xc-	(79)
	HCQ	LPS-treated monocytes	Not known yet.	Downregulation of ROS and MDA; upregulation of GSH and GPx	(80)
	CTX	Glioblastoma cells; breast cancer cells	Upregulation HMOX-1 to increase LIP	Not known yet.	(81)
Biological agents	ADA+IKE/ ETN+IKE	RA-FLSs	Not known yet.	Counteraction of TNF-induced cystine uptake and GSH synthesis to increase ferroptosis sensitivity	(39)
	TCZ	RA-FLSs	Counteraction of IL-6-induced upregulation of LIP to decrease ferroptosis sensitivity	Not known yet.	(39)
Anti-rheumatic	RES	RA-FLSs	Not known yet.	Activation of the Keap1-Nrf2 pathway	(82)
natural extracts	ICA	Synovitis cell model	Downregulation of TfR1 and NCOA4	Upregulation of GPX4, SLC7A11, SLC3A2L and NRF2	(38)
		MC3T3-E1 osteoblast cells	Not known yet.	Attenuation of iron-induced ROS production	(68)
		Iron overload mice	Attenuation of iron deposition	Not known yet.	(68)
	QUR	OA mouse model	Not known yet.	Downregulation of ROS; upregulation of GSH and GPx	(83)
	BA	GA mouse model	Not known yet.	Upregulation of GPx activity to reduce MDA	(57)
Other molecules	Gly	RA-FLSs	Methylation of GPX4 promoter; downregulation of FTH1	Not known yet.	(41)

ADA, adalimumab; BA, boswellic acid; CTX, cyclophosphamide; DMARDs, disease-modifying anti-rheumatic drugs; DPEP1, dipeptidase-1; DXMS, dexamethasone; ETN, etanercept; FLS, fibroblast-like synoviocyte; FTH1, ferritin heavy chain1; GSH, glutathione; HCQ, hydroxychloroquine; HMOX-1, heme oxygenase 1; IBU, ibuprofen; ICA, icariin; IKE, imidazole ketone Erastin; IND, indomethacin; Gly, glycine; LIP, LPS, lipopolysaccharide; MTX, methotrexate; NCOA4, nuclear receptor coactivator 4; NSAIDs, nonsteroidal anti-inflammatory drugs; PDAC, pancreatic ductal adenocarcinoma; QUR, quercetin; RES, resveratrol; ROS, reactive oxygen species; SASP,sulfasalazine; TfR1, transferrin receptor 1; TCZ, tocilizumab.

### 4.1 Glucocorticoids

As an important option in the bridge therapy and treatment of extra-articular symptoms of inflammatory arthritis, the role of glucocorticoid preparations can’t be ignored. Dexamethasone (DXMS) can induce ferroptosis *via* P53/SLC7A11/GPX4 pathway in pre-osteoblast MC3T3-E1 cells and osteocyte MLOY4 cells in glucocorticoid-induced osteonecrosis (75). DXMS could reduce GSH by upregulating the expression of dipeptidase-1 (DPEP1) to increase the sensitivity to ferroptosis in HT1080 cells, and prednisolone was found to have a similar effect on ferroptosis-sensitivity-increasing effect in the study (74).

### 4.2 Nonsteroidal anti-inflammatory drugs

As the basic therapeutic drug in the field of rheumatism, some of the nonsteroidal anti-inflammatory drugs (NSAIDs) have been linked to ferroptosis. Indomethacin (IND) was found to normalize the increased MDA and GPx levels in the sodium urate crystal-induced GA mouse model (57). In Gao et al., ibuprofen (IBU), a widely used NSAID, could induce ferroptosis in glioblastoma cells by downregulating of Nrf2 pathway to inhibit the expression of GPX4 and SLC7A11 (76).

### 4.3 Disease-modifying anti-rheumatic drugs

Among disease-modifying anti-rheumatic drugs (DMARDs), many have been shown to regulate ferroptosis, although few conclusions were drawn from studies of inflammatory arthritis. As the most commonly used DMARDs in RA, methotrexate (MTX) has been found to promote the production of ROS, and some researchers have suggested that its immunosuppressive effect might be related to the apoptosis of T cells induced by oxidative stress (77). In the MTX-treated hippocampal HT22 cell line, iron overload and ROS accumulation were observed, as well as increased FTH1 and decreased NCOA4 (78). Another DMARDs, sulfasalazine (SASP), commonly used in the treatment of chronic inflammation such as inflammatory bowel disease and arthritis, has been found to induce ferroptosis in glioma cells (84). In 2001, Gout et al. found that SASP inhibited lymphoma growth *in vivo* and *in vitro*, which was the first demonstration that it was an effective inhibitor of System Xc- (79). However, whether hydroxychloroquine (HCQ), an antimalarial widely used in rheumatic diseases as DMARDs, has the effect of regulating ferroptosis remains an open field of research. A study has shown that HCQ can reduce LPS-induced apoptosis by inhibiting the transient receptor potential vanilloid 1 (TRPV1) pathway/ROS signaling pathway in human monocytes (80). Notably, HCQ treatment reversed LPS-induced upregulation of MDA and cytoplasmic ROS and downregulation of GSH and GPx in monocytes, which had some implications for ferroptosis (80). Although cyclophosphamide (CTX) was not recommended in the treatment of RA, it was still the choice for cutaneous vasculitis accompanied by RA. In 2022, Shi et al. found that CTX could increase LIP and induce ferroptosis by upregulating heme oxygenase 1 (HMOX-1) expression in glioblastoma cells and breast cancer cells, as well as *in vivo (* 85). In the same year, other researchers proposed an alternative idea that CTX may lead to GPX4 degradation, which in turn leads to caspase-independent parthanatos rather than lipid peroxidation-mediated ferroptosis in human leukemia cell lines, which provided new perspectives on the role of GPX4 beyond ferroptosis (81).

### 4.4 Biological agents

As another important kind of treatment, biological agents have always been a hot spot in the field of rheumatology. According to our previous research, in RA patients screened by proteomic analysis, the only differentially expressed protein (DEPs) that was significantly changed between the non-responsors and responsors group after combination treatment of MTX + leflunomide (LEF) + infliximab (IFX) was Tf (86), which could be a clue that biological agents regulate ferroptosis. Until February 2022, an important study that demonstrated the role of many cytokines in regulating FLSs sensitivity to ferroptosis, directly certified the role of biological agents in regulating ferroptosis. In that paper, the researchers used TNF antagonists combined with IKE to induce ferroptosis of RA-FLSs, which reduced cartilage and bone damage in CIA mice, and they also used an IL-6 antagonist and a TGF-β antagonist to confirm the ferroptosis-regulating function of these two cytokines (39). As the researcher stated in the paper, since cytokines have been shown to have the potential to modulate ferroptosis sensitivity, the combination of different biological agents with ferroptosis inducers or inhibitors to improve therapeutic efficacy warrants further investigation.

### 4.5 Natural extracts and others

Many natural extracts have been found to have anti-ferroptosic functions in RA, OA and GA. In RA, resveratrol (RES), a plant extract with various pharmacological effects, was considered a potential anti-rheumatic drug and found to induce the proliferation and migration of RA-FLSs by activating the Keap1-Nrf2 pathway, which is an important ferroptosis-regulating pathway in 2019 (82). In the same year, Jing et al. found that icariin (ICA), an active ingredient extracted from Epimedium, could prevent ROS production and mitochondrial dysfunction caused by iron overload *in vivo* and *vitro* osteoporosis model, although the authors attributed this effect to the fact that ICA could inhibit osteoblast apoptosis (68). Two years later, the function of ICA that inhibit ferroptosis in synoviocytes in the synovitis cell model was observed, thus ICA was considered a potential therapeutic agent for RA and OA (38). Similarly, another plant extract, quercetin (QUR), could alleviate disease severity by attenuating ROS generation, increasing the expression levels of GSH and GPx, and reversing mitochondrial dysfunction in the OA model mice (83). And in the GA mouse model, the researchers observed that boswellic acid (BA) had a similar function to IND in decreasing the MDA and GPx levels (57).

There was also a small molecule, glycine (Gly), which was found to enhance ferroptosis *via* S-adenosylmethionine (SAM)-mediated GPX4 promoter methylation and reduction of FTH in RA (41), but there is still a long way to go before it can be used as a drug for the treatment of RA.

## 5 Discussion

In the process of exploring the relationship between the pathogenesis of inflammatory arthritis and ferroptosis, we observed evidence of iron accumulation, lipid peroxidation, and mitochondrial dysfunction in tissues or cells of RA, OA, GA, and AS, and we focused on RA. However, the mechanism is still unclear (**Figure 2**), and it is worth further exploring whether ferroptosis is a cellular adaptive process or a manifestation of cellular dysfunction. According to the current studies, ferroptosis resistance of FLSs might be associated with synovial proliferation and many DMARDs including MTX, SASP and CTX have been shown to have pro-ferroptotic effects, so the use of ferroptosis inducers to relieve joint symptoms may be a promising strategy in inflammatory arthritis patients. However, many natural extracts, including RES, ICA, QUR, and BA, have been shown to inhibit ferroptosis. Meanwhile, since ferroptosis is pro-inflammatory and iron overload and ROS can lead to cartilage and bone destruction (42, 68), how to balance the induction of ferroptosis targeting FLSs with the inhibition of ferroptosis in other cells is a big challenge.

The relationship between ferroptosis in synoviocytes and ferroptosis in other cells is also questionable. For example, macrophages could release TNF to bind different TNF receptors (TNFR) on FLSs to affect the ferroptosis sensitivity of different subpopulations of FLS. Long-term TNF exposure could increase the ferroptosis sensitivity of FLSs through TNFR2 (39). Activation of M1 macrophages, which are the proinflammatory state of macrophages and can secrete proinflammatory cytokines, was related to the high intracellular iron status (87). The proinflammatory effect of macrophages appears to be closely related to ferroptosis, so ferroptosis inhibitors may be used to inhibit macrophage ferroptosis to perform therapeutic effects in inflammatory arthritis. As for chondrocytes, it seemed to be a consensus for many years that high levels of ROS could lead to inhibition of chondrocyte activity, leading to cartilage damage (88). In addition to inhibiting the effect of growth factors on chondrocytes (26), ROS could also activate MMP to promote chondrocyte apoptosis (89). Even more, by participating in the formation of pannus which is one of the key events in the pathogenesis of RA, angiogenesis could be activated by inflammation, immune imbalance, and hypoxia (90). Besides, ferroptosic cells could release DAMPs such as HMBG1 which has been proven to be a pro-angiogenic factor in cancer, cornea neovascularization, and ischemia-induced angiogenesis (91). Therefore, whether ferroptosis in synoviocytes can affect vascular endothelial cells may also be a question worth considering.

## 6 Conclusion

In this review, we summarized studies of ferroptosis in various inflammatory arthritis, some of which could only be called clues, and we hope that they will serve as evidence to inspire follow-up studies. At the same time, we listed some anti-rheumatic drugs and natural extracts that may have potential functions for regulating ferroptosis, and most of them still need to be studied and confirmed in more depth. The current study of ferroptosis in inflammatory arthritis is obviously in its infancy, and we have full confidence and believe in its promising future.

## Author contributions

SC and MT prepared the manuscript. FL, BZ, and DX conceived the idea and reviewed the drafts, and provided important information for the completion of this manuscript. All authors contributed to the article and approved the submitted version.

## Funding

This study was supported by Natural Science Foundation of HUNAN province (2021JJ30934); Hunan Provincial Health Committee 225 Talent Project (2019–196); Educational Fund of Hunan Provincial Finance Department (2021–22–2050205).

## Conflict of interest

The authors declare that the research was conducted in the absence of any commercial or financial relationships that could be construed as a potential conflict of interest.

## Publisher’s note

All claims expressed in this article are solely those of the authors and do not necessarily represent those of their affiliated organizations, or those of the publisher, the editors and the reviewers. Any product that may be evaluated in this article, or claim that may be made by its manufacturer, is not guaranteed or endorsed by the publisher.
